# Home-based phototherapy for neonatal hyperbilirubinemia: A one-time Canadian Paediatric Surveillance Program Survey

**DOI:** 10.1093/pch/pxae045

**Published:** 2025-04-17

**Authors:** Karissa Young, Charlotte Moore Hepburn, Michael Miller, Farah Abdulsatar

**Affiliations:** Department of Paediatrics, Schulich School of Medicine and Dentistry, Western University, London, Ontario, Canada; Department of Paediatrics, The Hospital for Sick Children, Toronto, Ontario, Canada; Department of Paediatrics, Schulich School of Medicine and Dentistry, Western University, London, Ontario, Canada; Children’s Health Research Institute, London, Ontario, Canada; Department of Paediatrics, Schulich School of Medicine and Dentistry, Western University, London, Ontario, Canada; Children’s Health Research Institute, London, Ontario, Canada

**Keywords:** *Adverse events*, *Canadian Paediatric Surveillance Program*, *Home phototherapy*, *Neonatal hyperbilirubinemia*, *Paediatrics*

## Abstract

**Objectives:**

Home-based phototherapy (HP) has gained traction as an alternative to hospital-based treatment for neonatal hyperbilirubinemia, but the safety of this practice remains unclear. This study aimed to identify adverse events (AEs) associated with HP in Canada.

**Methods:**

A one-time survey, distributed through the Canadian Paediatric Surveillance Program, collected retrospective data from paediatricians. The survey included questions about the use of HP, AEs associated with HP, potential HP AE risk factors, and outcomes. A descriptive statistical analysis was conducted.

**Results:**

The survey response rate was 31% (844/2741), with 497 respondents indicating that they provide care for neonatal hyperbilirubinemia. Among those 497 respondents, 58 (12%) reported working at a centre that provides HP. AEs were reported by 15 (3%) physicians with 21 cases of AEs associated with HP in the preceding 12 months. Most AEs resulted in admissions or readmissions to the hospital for inpatient phototherapy. No serious AEs or long-term consequences were reported. Risk factors were identified in 67% of cases, with infant-related factors identified more frequently than provider, system, or family-related factors. Formal protocols for patient assessment and follow-up were in place at most centres that provide HP.

**Conclusion:**

This survey revealed no serious AEs related to HP, as reported by paediatricians in Canada, in the preceding 12 months. The survey also revealed that while HP is available in Canada, there is limited access and a lack of standardization to its administration. This study provides valuable insights into the safety and practice of HP for neonatal hyperbilirubinemia in Canada.

## BACKGROUND

Hyperbilirubinemia is the most common reason for hospitalization in infants aged less than 10 days (1,2). It is estimated that approximately 60% to 80% of newborns develop neonatal hyperbilirubinemia, with approximately 2% requiring medical intervention (3,4). The current standard treatment involves phototherapy administered in a hospital setting (3,5). However, advancements in technology have led to the development of portable and compact options like fiberoptic phototherapy systems (FPS) (6). These advancements have revolutionized phototherapy, offering an efficient and easy-to-use option for patients and their families to employ outside the hospital setting. Additionally, FPS allows for uninterrupted breastfeeding and minimal separation of the mother-infant dyad, in turn promoting bonding and nurturing this important parent–infant relationship (7). These advancements in FPS technology, combined with their patient- and system-level benefits, have significantly improved the treatment of neonatal hyperbilirubinemia, paving the way for home-based phototherapy (HP).

While HP has been widely utilized in the USA for more than two decades (8–12), the 2022 American Academy of Pediatrics (AAP) guidelines take a cautious approach in recommending the use of HP in infants, with strict exclusion of those with neurotoxicity risk factors. Furthermore, the guidelines highlight the importance of checking daily total serum bilirubin (TSB) levels as a crucial safety intervention (4). The efficacy of HP remained inconclusive as a 2014 Cochrane review found insufficient studies meeting the necessary selection criteria to draw definitive conclusions (13). However, the COVID-19 pandemic has promoted a shift towards delivering hospital care at home for various illnesses when feasible (14). One particular form of home-based hospital care that has gained attention is the assessment of bilirubin levels in newborns at home (15–17). In parallel, the adoption of HP has been growing worldwide and gaining considerable traction (18,19). HP has been recognized for its cost-effectiveness, reduction in healthcare system expenses (20,21), enhancement of parent–infant bonding, and the reduction of disruptions to that relationship (22,23). In terms of safety, studies have shown that less than 5% of low-risk neonates receiving HP require hospitalization during their home treatment (20,24), and no neonates in these studies required exchange transfusion (ET), a treatment reserved for critically elevated TSB (3). Importantly, there has been one case report of a neonate who, after receiving treatment with in-hospital phototherapy followed by HP, required ET and developed acute bilirubin encephalopathy (ABE). This neonate had ABO incompatibility, and initially tested negative for direct antiglobulin (DAT), as well as spherocytosis. Genetic testing showed that the neonate had a gene mutation predicted to impact erythrocyte cytoskeletal protein function. The timing of follow-up, 44 h after discharge from hospital on HP, also likely contributed to the severity of this adverse event (AE) (25). Learning from case reports of rare, but serious, AEs allows for the optimization of HP protocols to ensure appropriate candidate selection, safety monitoring, and follow-up care.

At present, HP is available in some regions in Canada, yet standardization, both in terms of access and protocols, is lacking across the country (26). Given the evolving landscape of neonatal hyperbilirubinemia treatment with the widespread use of HP internationally, it is imperative to assess HP’s safety profile in the Canadian context. The primary objective of this study was to identify AEs related to HP within Canada over the past 12 months. Secondary objectives included identifying risk factors associated with these AEs and gathering information to describe the current status of HP in Canada.

## METHODS

A one-time survey, distributed through the Canadian Paediatric Surveillance Program (CPSP), collected retrospective information from paediatricians about HP for neonates with unconjugated hyperbilirubinemia. The CPSP is a joint program of the Canadian Paediatric Society and the Public Health Agency of Canada designed to collect voluntary surveillance data from practicing paediatricians related to rare conditions or outcomes (27). The survey was distributed to the 2741 paediatricians who participated in the CPSP, was made available in both English and French and was delivered through email or paper reporting forms, according to participant preference. The survey was accessible from December 7, 2022, to January 17, 2023, with two reminders sent during this period.

Survey questions were developed by the authors in collaboration with CPSP Steering Committee stakeholders and paediatricians, following an iterative approach (see Supplementary Appendix). A beta test of the survey was conducted at the Children’s Hospital in the London Health Science Centre, with feedback obtained from 15 participating paediatricians.

Survey respondents were initially asked for basic demographic data, as well as physician involvement in the care of neonates with unconjugated hyperbilirubinemia. They were then asked about the availability of HP for neonates at their respective centres, and if applicable, additional questions were posed regarding HP provision. Further questions focused on experiences with AEs associated with HP in the last 12 months, including cases that required hospitalization for inpatient phototherapy, ET, the development of ABE, and neonatal deaths. Respondents who reported one or more AEs were requested to provide details, including the number of cases, the specific type of AE, associated risk factors, and the outcomes. Risk factors were categorized as infant risk factors (e.g., sepsis, significant weight loss, DAT positivity, glucose-6-phosphate dehydrogenase [G6PD] deficiency), provider or system factors (e.g., inappropriately delayed scheduled follow-up, unable to secure timely follow-up laboratory results, lack of/incomplete screening for risk factors for hyperbilirubinemia, technology issue/device malfunction), and family factors (e.g., parent-delayed follow-up, poor treatment compliance, insufficient parent/caregiver teaching, social risk factors).

The statistical analysis was descriptive in nature. Categorical responses were summarized using frequencies and percentages. SPSS v.28 (IBM Corp., Armonk, NY) was used for all analyses. The study was funded through a CPSP in-kind surveillance grant awarded to the authors. As anonymous information was collected by the CPSP, it was exempt from the institutional research ethics board as per TCPS2 Article 2.4 and a letter was obtained to that effect. To maintain confidentiality, results under n = 5 were suppressed, as per CPSP policies.

## RESULTS

The survey response rate was 31% (844/2741). Of the survey respondents, 59% (497/844) reported that they provided care for neonates with hyperbilirubinemia. Characteristics of the physicians who provide care to neonates with hyperbilirubinemia are included in Table 1. Three surveys included AEs that were excluded because the respondent reported cases outside of the last 12-month window. When there was a discrepancy between the reported number of cases and the number of cases reported with accompanying details, we included only the cases reported with details in our total case count. For the respondents who cared for newborns with hyperbilirubinemia (n = 497), 0.9% of the total data elements were missing.

**Table 1.** Characteristics of paediatricians respondents who provide care to neonates with hyperbilirubinemia

Characteristics	n (%)
Type of physician*	
General paediatrician	392 (79)
Neonatologist	50 (10)
Emergency physician	36 (7)
Subspecialist	22 (4)
Missing/Other	^†^
Practice type*	
Urban	349 (70)
Suburban	103 (21)
Rural/remote	73 (15)
Missing	14 (3)
Practice type*	
Academic	264 (53)
Non-academic	205 (41)
Missing	60 (12)
Practice setting*	
Inpatient	322 (65)
Outpatient/community	287 (58)
ED/urgent care	154 (31)
NICU/PICU	187 (38)
Missing	39 (8)

*Counts and percentages may be larger than the number of respondents due to multiple responses per respondent;

^†^Under five respondents.

### Status of HP in Canada

HP was offered at the centres of 12% (58/497) of respondents who provide care for newborns with unconjugated hyperbilirubinemia. The practice settings of those respondents were reported as 67% (39/58) urban, 19% (11/58) suburban, and 22% (13/58) rural/remote, with some respondents reporting more than one practice setting. Among respondents who had HP available at their centres, 60% (35/58) said they had formalized protocols to assess patient appropriateness for HP and 83% (48/58) had a formal process or protocol to ensure appropriate patient follow-up. Respondents reported that HP programs generally provided support to families, with 41% (24/58) reporting 24/7 support and 22% (13/58) reporting part-time support (e.g., during regular business hours Monday to Friday). No dedicated support outside of planned follow-up was reported by 16% (9/58) of respondents.

### AEs associated with HP

Of the 497 respondents who reported providing care for neonates with unconjugated hyperbilirubinemia, only 15 (3%) reported having cared for a patient with an AE associated with HP in the preceding 12 months with a total of 21 cases of AE identified. The type of AE was reported in 20/21 (95%) cases, with the vast majority (19/20, 95%) being admissions/readmissions to hospital for inpatient phototherapy. There were no serious AEs reported, including no cases of ABE, neonates requiring intravenous immunoglobulin (IVIG), or ET. In the 15 cases with a reported outcome, there were no anticipated permanent sequelae related to the AE. Risk factors were identified in 67% (14/21) of cases of AEs associated with HP. Most of the risk factors identified were infant-related (8/14, 57%). Provider or system factors and family factors were each identified in fewer than five cases.

## DISCUSSION

The findings of this study offer significant insights into the safety and practices associated with HP for neonatal hyperbilirubinemia in Canada. Among the 497 respondents providing care for neonatal hyperbilirubinemia, 21 cases with AEs associated with HP were reported over the previous 12 months. This aligns with previous studies that have found a minority of infants treated with HP require admission/readmission for inpatient phototherapy. Despite the number of AEs, none were severe, and no cases were anticipated to have permanent sequelae. This builds on previous studies demonstrating the safety of HP and provides data from a heterogeneous population representing a variety of different ways in which HP is provided and monitored, increasing the external validity and better representing the safety of HP in Canada as it is currently being provided (20,24).

Among cases with AEs, infant factors were the most commonly reported risk factors, differing from previous findings by Pettersson et al. (24), where all hospitalizations were attributed to poor treatment compliance. This discrepancy may be attributed to the stringent criteria for infants eligible for HP in Pettersson’s study with criteria that excluded infants who were DAT positive, had over 10% weight loss from birth weight, or developed hyperbilirubinemia at under 48 h of age (23). Infants had unconjugated bilirubin level that required phototherapy and was more than 25 μmol/L away from the level that would require ET as per Swedish guidelines (23). In Chang’s and Waite’s study, where HP was provided at the discretion of the referring clinician, a low rate of hospitalization was also reported (20). Although they had high-risk patients, only 28% were at or above the phototherapy treatment threshold at the initiation of HP. This is likely related to a previous AAP recommendation that HP should only be provided to infants with a bilirubin level 35 to 50 μmol/L below the treatment guideline with the new guideline published in 2022 outlining that HP can be used for infants who have a bilirubin level up to 17 μmol/L above the treatment threshold (4,28). Their study indicated that a higher TSB level at HP initiation was the sole risk factor associated with subsequent hospitalization, with no significant impact from other infant factors they examined (20). Christensen et al. (24) published a case report of a neonate who required ET after receiving HP, who had multiple infant factors including ABO incompatibility and spherocytosis which were identified retrospectively.

Our findings also shed light on the current state of HP availability across regions and practice settings in Canada. While most respondents reported centres offering HP were located in urban settings, this aligns with the demographic distribution of respondents. In contrast, respondents working in rural communities were more likely to report access to HP centres. Moreover, a mere 60% of the centres with access to HP have implemented a structured protocol for evaluating the suitability of neonates for this intervention. The establishment of round-the-clock medical support, coupled with the presence of formalized protocols, can enhance the confidence of both healthcare providers and families in choosing this treatment option (22).

Despite the valuable contributions of this study regarding HP for neonatal hyperbilirubinemia, certain limitations should be acknowledged. The relatively low response rate of 31% introduces the potential for response bias, and the sample size may limit the generalizability of the findings. It is also important to note that this survey focused solely on paediatricians, while many well newborns with hyperbilirubinemia are managed by family physicians, midwives, or other health practitioners potentially resulting in missed cases. Differences may also exist in HP programs provided by paediatricians in this study compared to those offered by other health practitioners. To address these limitations, future research could employ larger sample sizes and prospective study designs for a more comprehensive evaluation of HP’s safety and effectiveness.

## CONCLUSION

This study contributes to our understanding of the safety and utilization of HP for neonatal hyperbilirubinemia in Canada. The findings reveal a low number of AEs associated with HP, with no reports of severe AEs or long-term sequelae in infants cared for in the preceding 12 months of this survey. There is a need for ongoing efforts to establish evidence-based guidelines that can further enhance the safety and efficacy of HP to optimize neonatal care for hyperbilirubinemia and foster the widespread adoption of HP as a standard of care across Canada.

## SUPPLEMENTARY DATA

Supplementary data are available at *Paediatrics & Child Health* Online.

pxae045_suppl_Supplementary_Material
