# Supplementary material for: Home-based phototherapy for neonatal hyperbilirubinemia: A one-time Canadian Paediatric Surveillance Program Survey
Source: Paediatr Child Health. 2025 Apr 17;30(4):279–83. doi: 10.1093/pch/pxae045 (PMC12316525; doi:10.1093/pch/pxae045)
Supplement: pxae045_suppl_Supplementary_Material [file pxae045_suppl_supplementary_material.pdf]

CPSP
CANADIAN PAEDIATRIC SURVEILLANCE PROGRAM

Survey
Home-based phototherapy

Your contribution is greatly appreciated.

1. Do you care for newborns with unconjugated hyperbilirubinemia? ❍ Yes ❍ No

   ***If No,* thank you for completing this survey.**

2. Which of the following best describes your practice?
   ❍ General paediatrician ❍ Paediatric subspecialist; specify: ________________ ❍ Other, specify: ____________________

3. What are the first 3 digits of your practice's postal code? ___ ___ ___

4. Type of practice setting (select all that apply):
   a) ❑ Urban ❑ Suburban ❑ Rural/remote
   b) ❑ Academic ❑ Non-academic
   c) ❑ Neonatal/paediatric intensive care unit ❑ Inpatient hospital ❑ Emergency/urgent care centre
   ❑ Outpatient clinic/community setting

5. At present, do you, or does your centre, offer home-based phototherapy for patients with unconjugated hyperbilirubinemia?
   ❍ Yes ❍ No ❍ Unknown ***If No or Unknown*, proceed to question 6. *If Yes:***
   5.1 Is there a formal protocol to assess patient appropriateness for home-based phototherapy? ❍ Yes ❍ No ❍ Unknown
   5.2 Is there a formal process or protocol to ensure appropriate patient follow up? ❍ Yes ❍ No ❍ Unknown
   5.3 Is there on-call support (staffed by physicians, nurse practitioners, nurses, or midwives) for parents/caregivers of neonates receiving home-based phototherapy?
   ❍ Yes, it is a 24/7 service
   ❍ Yes, support for parents/caregivers is available during regular business hours (Monday to Friday)
   ❍ Other, please specify:_____________________________
   ❍ No
   ❍ Unknown

6. In the past 12 months, have you cared for a patient with an adverse event (including admission/readmission) associated with home-based phototherapy for unconjugated hyperbilirubinemia?
   ❍ Yes, specify number of cases: ________ ❍ No

   ***If No*, thank you for completing this survey.**

7. For each patient with an adverse event(s) associated with home-based phototherapy in the last 12 months, complete the information below. If you have encountered more than two cases, contact the Canadian Paediatric Surveillance Program for an additional survey.

	**Case 1**	**Case 2**
Adverse event(s) (select all that apply)	❑ Admission/readmission for inpatient phototherapy<br>❑ Hyperbilirubinemia requiring intravenous immunoglobulin (IVIg)<br>❑ Hyperbilirubinemia requiring exchange transfusion<br>❑ Acute bilirubin encephalopathy<br>❑ Other, describe: _____________________________	❑ Admission/readmission for inpatient phototherapy<br>❑ Hyperbilirubinemia requiring intravenous immunoglobulin (IVIg)<br>❑ Hyperbilirubinemia requiring exchange transfusion<br>❑ Acute bilirubin encephalopathy<br>❑ Other, describe: _____________________________
Patient sex	❍ Female ❍ Male ❍ Intersex ❍ Unknown	❍ Female ❍ Male ❍ Intersex ❍ Unknown
Birth gestation	❍ ≤ 36 weeks ❍ 37 weeks ❍ ≥ 38 weeks ❍ Unknown	❍ ≤ 36 weeks ❍ 37 weeks ❍ ≥ 38 weeks ❍ Unknown
Who initiated home phototherapy?	❍ Midwife<br>❍ Nurse practitioner<br>❍ Family physician<br>❍ Paediatrician<br>❍ Other, specify: __________________<br>❍ Unknown	❍ Midwife<br>❍ Nurse practitioner<br>❍ Family physician<br>❍ Paediatrician<br>❍ Other, specify: __________________<br>❍ Unknown

Risk factor(s) for adverse event(s) (select all that apply)	❑ Sepsis<br>❑ Significant weight loss (≥ 15%) since birth<br>❑ Direct antibody test (DAT) positivity<br>❑ Glucose-6-phosphate dehydrogenase (G6PD) deficiency<br>❑ Parent-delayed follow-up<br>❑ Inappropriately delayed scheduled follow-up<br>❑ Unable to secure timely follow-up laboratory results (e.g., weekend/holiday delays)<br>❑ Lack of/incomplete screening for risk factors for hyperbilirubinemia<br>❑ Technology issue/device malfunction<br>❑ Poor treatment compliance<br>❑ Insufficient parent/caregiver teaching<br>❑ Social risk factors (e.g., housing insecurity, limited transportation, language barrier)<br>❑ Other, describe:____________________________<br>❑ None	❑ Sepsis<br>❑ Significant weight loss (≥ 15%) since birth<br>❑ Direct antibody test (DAT) positivity<br>❑ Glucose-6-phosphate dehydrogenase (G6PD) deficiency<br>❑ Parent-delayed follow-up<br>❑ Inappropriately delayed scheduled follow-up<br>❑ Unable to secure timely follow-up laboratory results (e.g., weekend/holiday delays)<br>❑ Lack of/incomplete screening for risk factors for hyperbilirubinemia<br>❑ Technology issue/device malfunction<br>❑ Poor treatment compliance<br>❑ Insufficient parent/caregiver teaching<br>❑ Social risk factors (e.g., housing insecurity, limited transportation, language barrier)<br>❑ Other, describe:____________________________<br>❑ None
Outcome (select all that apply)	❑ No anticipated permanent sequelae<br>❑ Suspicion for permanent sequelae<br>❑ Confirmed disability<br>❑ Changes on brain magnetic resonance imaging<br>❑ Other, specify:_____________________________<br>❑ Death<br>❑ Unknown	❑ No anticipated permanent sequelae<br>❑ Suspicion for permanent sequelae<br>❑ Confirmed disability<br>❑ Changes on brain magnetic resonance imaging<br>❑ Other, specify:_____________________________<br>❑ Death<br>❑ Unknown

Principal investigators: Karissa Holyer, Farah Abdulsatar
Co-investigators: David Millar, Michael Miller, Michael Sgro, Sepideh Taheri

Please return this survey with your monthly reporting form.
Thank you for your participation.

11/2022
